# Mesenchymal stem cell cryopreservation with cavitation-mediated trehalose treatment

**DOI:** 10.1038/s44172-024-00265-6

**Published:** 2024-09-09

**Authors:** Carla V. Fuenteslópez, Michael Gray, Simge Bahcevanci, Alexander Martin, Cameron A. B. Smith, Constantin Coussios, Zhanfeng Cui, Hua Ye, Viorica Patrulea

**Affiliations:** 1 Institute of Biomedical Engineering, Department of Engineering Science, University of Oxford, Oxford, UK; 2 Institute of Diagnostic and Interventional Radiology, University Hospital Zurich, Zurich, Switzerland; 3 Institute of Pharmaceutical Sciences of Western Switzerland, University of Geneva, Geneva, Switzerland; 4 School of Pharmaceutical Sciences, University of Geneva, Geneva, Switzerland

**Keywords:** Membrane biophysics, Biomedical engineering

## Abstract

Dimethylsulfoxide (DMSO) has conventionally been used for cell cryopreservation both in research and in clinical applications, but has long-term cytotoxic effects. Trehalose, a natural disaccharide, has been proposed as a non-toxic cryoprotectant. However, the lack of specific cell membrane transporter receptors inhibits transmembrane transport and severely limits its cryoprotective capability. This research presents a method to successfully deliver trehalose into mesenchymal stem cells (MSCs) using ultrasound in the presence of microbubbles. The optimised trehalose concentration was shown to be able to not only preserve membrane integrity and cell viability but also the multipotency of MSCs, which are essential for stem cell therapy. Confocal imaging revealed that rhodamine-labelled trehalose was transported into cells rather than simply attached to the membrane. Additionally, the membranes were successfully preserved in lyophilised cells. This study demonstrates that ultrasonication with microbubbles facilitated trehalose delivery, offering promising cryoprotective capability without the cytotoxicity associated with DMSO-based methods.

## Abstract (web summary)

The team led by Dr. Patrulea propose a method to use trehalose as a non-toxic cryopreservation & lyophilisation agent. They demonstrate how ultrasound & microbubbles can be used to successfully internalise trehalose into mesenchymal stem cells, preventing cryodamage.

## Introduction

The preservation of cells, tissues, and other biological samples is essential for basic and clinical research in fields such as bioengineering, medicine, and biology. However, this is not a trivial feat. Cryopreservation, which relies on using very low temperatures, is the most widely method used for prolonged cell storage and banking with a minimal loss in cell function^1^. Cells are usually stored in cryosoups, which are cryoprotectant solutions in which cells are suspended during the freezing stage, which are often made of 10% DMSO with 90% foetal bovine serum (FBS)^2^. Beyond cell storage, cryopreservation is essential not only for basic research but also for tissue and organ preservation^3^. However, freezing leads to extra-cellular ice (re)crystallisation, intracellular ice nucleation, cell dehydration, and osmotic shock, all of which damage the cells and can lead to cell death.

Cryopreservation-induced damage can be mitigated using permeating and/or non-permeating cryoprotectants (CPAs), also named small molecular weight or penetrating and high molecular weight or non-penetrating agents^4^. Permeating CPAs, including dimethylsulfoxide (DMSO) and glycerol, can cross cell membranes while non-permeating CPAs, such as poly(vinyl pyrrolidone), hydroxyethyl starch, poly(ethylene glycol), sucrose, raffinose, and trehalose remain outside the cell. Their specific mechanism of cryoprotection has been described by Elliott et al.^4^, although it is not fully understood. The emphasis is put mostly on their multimodal action arising from the combination of factors including hydrogen bonding between water and CPA, CPA-membrane interaction, formation of a polymer glassy matrix, and increase in CPA’s viscosity at low temperatures^4^.

Two of the most commonly employed CPAs, DMSO and glycerol^3^, are cytotoxic and are therefore suboptimal. Given the ability of permeating CPAs to reach intracellularly within a micromolar scale, it is expected that their toxic effects can be identified during long-term exposures; for instance, epigenetic changes in hepatic microtissues^5^, seizures, cardiac arrest^6^, growth inhibition and occasionally cell dysfunction^7^ and differentiation in embryonic stem cells^8^. Glycerol, although less toxic than DMSO, requires careful removal using a specialised deglycerolisation machine when cryopreserving red blood cells for transfusion^1^.

Trehalose is a biocompatible and natural disaccharide that can act as an osmoprotectant during dehydration and/or freezing, preventing the cell damage provoked by ice crystal formation^8,9^.

The protection mechanism of trehalose relies on the interaction of the –OH groups in trehalose with the polar head groups of phospholipids of the cell membranes, which stabilise the membranes^10^. By providing these hydrogen bonding groups to replace water molecules, proteins and biological structures can be stabilised^11^. However, the strength of the hydrogen bonds plays a crucial role, as a bond that is too strong may induce cell hydration disruption^12^.

Thus, it has the potential to prevent cell damage arising from ice crystal nucleation. Moreover, trehalose has been assessed and approved by the U.S. Food and Drug Administration (FDA) for use in food, vaccines, and protein preservation^9^.

To achieve successful cell cryopreservation, trehalose must be present on both sides of the cell membrane^3^. Trehalose can protect cells in a dehydrated state at adequate concentration. However, mammalian cells lack receptors that facilitate trehalose crossing their cell walls, necessitating additional mechanisms or modifications for this process^1^. Attempts to circumvent this challenge include strategies such as osmotic stress and rapid temperature changes, yet these methods have not been successful in maintaining cell function^9^. A promising alternative method is the application of ultrasound (US) in the presence of micron-scale bubbles, where stresses induced by oscillations of nearby microbubbles have been shown to open cell membrane pores and enhance intracellular drug delivery^13,14^. US-mediated microbubble activity, known as *cavitation*, may lead to a broad spectrum of bioeffects which are dependent on the applied US parameters including frequency, peak negative pressure, and exposure duration, as well as on the properties of the microbubbles themselves^15,16^. Temporary membrane poration with high cell viability has been achieved using relatively mild US parameters^17,18^ characterised by pressures no >0.5 MPa at frequencies in the order of 1 MHz. Microbubble acoustic radiation during US exposure can be used to noninvasively monitor and optimise the exposure process^19,20^, often by limiting broadband emissions associated with violent bubble collapses and potentially adverse bioeffects^21,22^.

The use of US for intracellular delivery of trehalose has previously been investigated for red blood cells^23,24^, using US frequencies ranging from 25 kHz to 8 MHz. Each study was conducted with a single trehalose concentration and, although the delivery mechanism was attributed to ultrasound-mediated microbubble activity, cavitation monitoring was uniformly absent. To our knowledge, there have been no peer-reviewed studies of US + microbubbles mediated cryopreservation performed with eukaryotic cells.

**Fig. 1:**
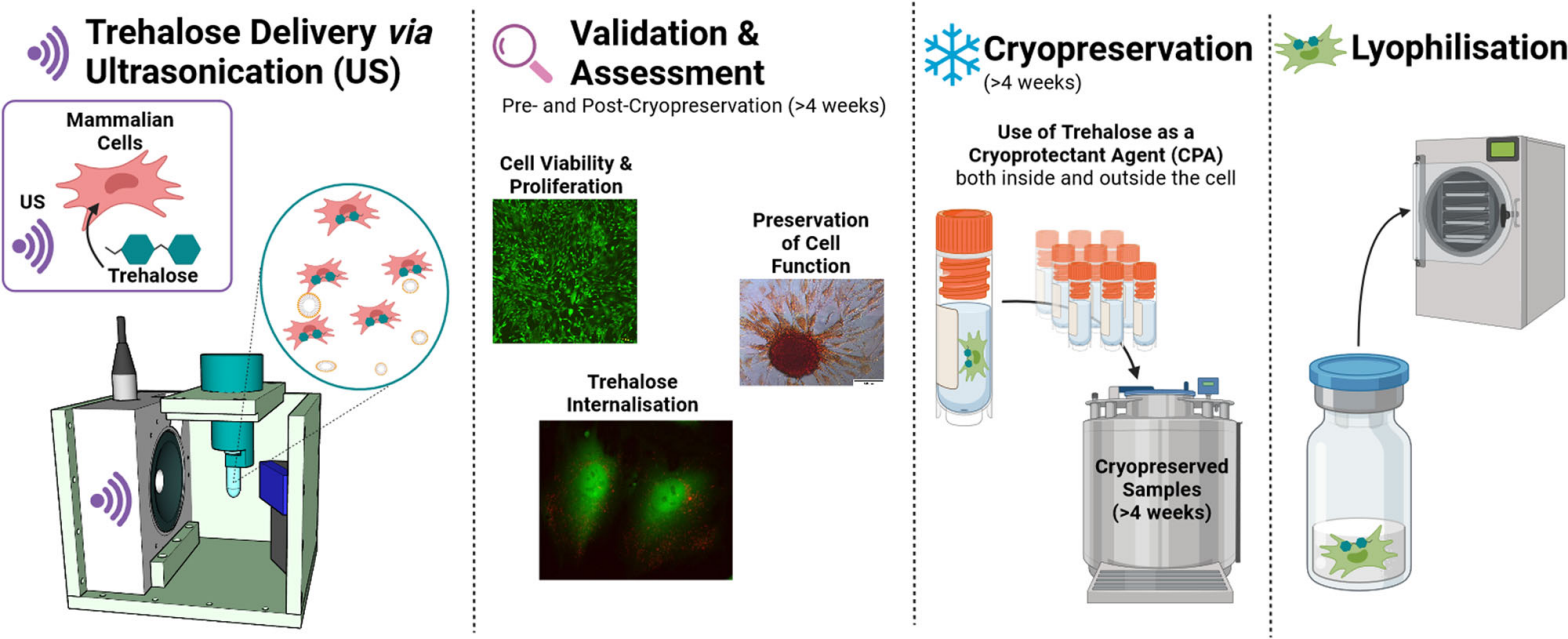
Overview of the development and validation of trehalose as a successful cryoprotectant agent (CPA) for mammalian cells. Trehalose delivered via US + microbubbles enable cryopreservation of at least 4 weeks while preserving cell viability, membrane integrity, and cell multipotency.

Here, we investigate the feasibility of using trehalose delivered via US  +  microbubbles (UMT) as a CPA and evaluate the trehalose concentrations best suited for the cryopreservation of mesenchymal stem cells (MSC) (Fig. 1). We demonstrate that the UMT process leads to intracellular trehalose, and further evaluate the suitability of US + microbubbles delivered trehalose pre- and post-cryopreservation, as well as post-lyophilisation in terms of cell viability and preservation of cell pluripotency. Additionally, we validate trehalose internalisation across the cell membrane and verify membrane integrity post-lyophilisation (Supplementary Materials).

## Methods

### Cell culture

Human MSCs, immortalised as described by Mihara et al.^25^, were a gift from the Department of Paediatrics and Adolescent Medicine, LKS Faculty of Medicine, The University of Hong Kong. MSCs were cultured in Dulbecco’s Modified Eagle Medium (1 g L^−1^ glucose; Gibco) media supplemented with 10% FBS (Gibco), 1% penicillin/streptomycin (Gibco Life Technologies, Grand Island, NY, USA), henceforth called DMEM, in a humidified incubator (37 °C, 5% CO_2_). Cell media was changed twice a week, and cells were passaged before reaching confluency.

### Ultrasonication device & parameters

**Fig. 2:**
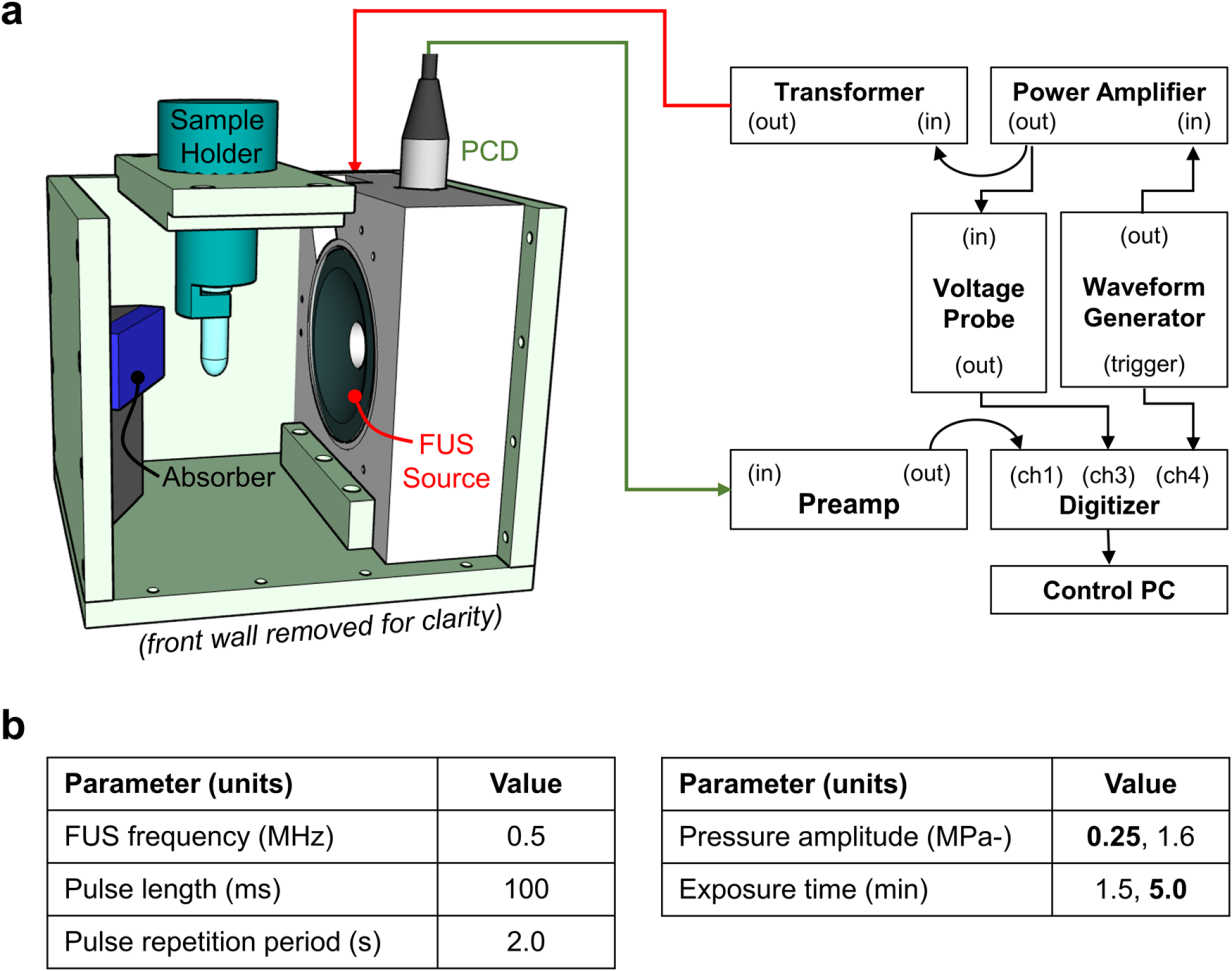
Ultrasound setup. Here, we, display the **a** ultrasound exposure chamber and supporting instrumentation and **b** ultrasound exposure conditions. Multiple ultrasound exposure conditions were used, the most commonly used ones are presented in bold.

Ultrasound exposures were carried out in a compact exposure chamber, with a focused ultrasound (FUS) source and a separate passive cavitation detector (PCD) housed in a single mounting block as illustrated in Fig. 2. A 500 kHz FUS source (H107, Sonic Concepts, Bothell, WA, USA) provided a pressure field with full-width half maximum dimensions of 4.1 and 25.2 mm in the lateral and axial directions, respectively. The source drive signal was supplied by a function generator (33250A, Agilent Technologies, Cheshire, UK), passed to a power amplifier (E&I 325LA, Rochester, NY, USA), then on to a matching transformer and the source transducer itself. Source field dimensions and input voltage–output pressure relationships were determined in free-field through calibration with a needle hydrophone (NH-200, Precision Acoustics, Dorchester, UK).

A single-element broadband transducer (V309, 5 MHz, 6.35-mm radius, Olympus NDT, Essex, UK) was coaxially aligned with the source using a 45° mirror (F102, Olympus) to serve as a PCD for monitoring emissions from the exposed sample. The signal output was amplified (SR445A, Stanford Research Systems, Palo Alto, CA, USA) and terminated with a 50 Ω shunt for recording. PCD and power amplifier output signals were monitored with a USB-based 14-bit digitiser (HS6-diff, TiePie Engineering, Sneek, Netherlands).

PCD data were processed to determine spectral content as a function of time allowing assessment of the type and level of microbubble activity in the exposed samples. PCD power spectral density as a function of sample exposure time was calculated using Welch’s method, employing 50% window overlap and a 5 kHz frequency step size (10^−2^ of the drive frequency).

The exposure chamber was filled with filtered and degassed water maintained at 35 ± 1 °C using a submersible heater (Aquael 50W, Warsaw, Poland) and with the application of insulation around the exterior chamber walls. An absorber panel (Aptflex F28, Precision Acoustics) was installed opposite the source for reverberation control. Samples containing a combination of cells, microbubbles, and cryoprotectants were placed in 2.0 ml Eppendorf or 1.8 ml cryotubes and were suspended in the focus of the FUS source using purpose-built holders.

The list of evaluated exposure conditions is provided in Fig. 2b. Most experiments were performed with 0.5 MHz frequency, 100 ms pulse length, 2 s pulse repetition period, 0.25 MPa peak negative pressure, and a 5-min exposure time. The frequency, pulse time scales, and pressure were based on prior studies of microbubble-mediated drug delivery^26–28^. The reduction from 5 to 1.5-min exposure time supported an investigation of treatment throughput, while the increase in pressure to 1.6 MPa was made to assess uptake and viability under conditions that have been used to enhance drug transport^29^.

### Trehalose delivery via ultrasonication

Trehalose powder (D-(+)-trehalose dihydrate) was a kind donation from Pfanstiehl, Switzerland. Cell suspensions were prepared in a 2.0 ml Eppendorf tube (ThermoFisher) at a cell density of 1 × 10^6^ cells ml^−1^, containing trehalose at 0, 50, 100, 250, 500, 750, and 1000 mM (in DMEM without phenol red) and 1% (v/v) SonoVue microbubbles (Bracco, Milan, Italy) as a cavitation agent. SonoVue (SF6 gas in a lipid shell, 1–11 μm size) was reconstituted according to the manufacturer’s instructions.

Then, the tube was transferred into the US chamber and treated as described in the previous section. Following treatment, cells were centrifuged for 5 min at 1500× *g*, and the supernatant was collected and replaced by DMEM (with 10% FBS). Cells were left for incubation at 37 °C and 5% CO_2_ for an additional 30 min to accommodate trehalose loading and allow pore membranes closure before counting cells with Trypan blue 0.4% (Invitrogen, Eugene, OR, USA).

The following two controls were also prepared: control (cells) are cells cultured under standard conditions without exposure to US or trehalose and control (cells + US) are cells exposed to US + microbubbles but not treated with trehalose. A summary of experimental and control cells outlining the treatment received is outlined in Table S1.

### Cryopreservation

Cells were cryopreserved (slow freezing) for at least 4 weeks at −195 °C in liquid nitrogen in one of two cryosoups: 10% DMSO + 90% FBS (standard practice for MSCs) and 5% trehalose in DMEM + 2.5% dextran in DMEM. A comparison of the composition of the cryosoups is included in the Supplementary Materials (Table S2). Following cryopreservation, cells were thawed, and a range of experiments were run to validate a successful cryopreservation through cell viability and cell differentiation assays.

### Cell viability

Once treated with UMT, cells were seeded (density: 5 × 10^4^ cells ml^−1^, 100 μl well^−1^) in 96-well plates in triplicates (UMT low/high pressure and cryosoup comparisons) or quadruplicates (UMT exposure time). Media was changed every 3 days. On days 3 and 5 post-seeding, cell media was removed and 100 μl of the Cell Counting Kit-8 (CCK-8; Dojindo, Japan) was added to each well. Cells were incubated for 1.5 h before measuring the absorbance at 450 nm using a microplate reader (SpectraMax i3x, Molecular Devices LLC, San Jose, CA, USA) and its software (SoftMax Pro; Version 7.0, Molecular Devices LLC, San Jose, CA, USA). The data were then processed to obtain the fold increase for each datapoint using the average of the respective control (cells) at day 3 value as a baseline.

Additionally, microscopy images of cells were captured after UMT treatment, both pre- and post-cryopreservation. Experimental and control samples were imaged using a fluorescence microscope, staining live (Calcein-AM; green) and dead (ethidium homodimer-1; red) cell using a Live/Dead Viability Kit (Molecular Probes Invitrogen, Eugene, OR, USA) according to the manufacturer’s instructions. Briefly, cell media was removed, and three PBS washes were done, before adding a solution comprising of an EthD-1 (20 μl of a 2 mM solution), Calcein-AM (5 μl of a 5 mM solution), and PBS (10 ml). Cells were incubated for 30 min at room temperature, away from the light, and imaged with a fluorescence microscope using red and green channels.

### Adipogenic differentiation assay

Cells previously treated with UMT were seeded in 48-well plates (1 × 10^4^ cells cm^−2^) and incubated with differentiation media (MesenCult™ Adipogenic Differentiation Kit (Human), StemCell Technologies, Canada, Vancouver BC) and followed the manufacturer’s protocols. Triplicates were prepared and media was changed every 3 days. MSCs were imaged after 14 days with a contrast-phase microscope after cells had been washed with PBS (3×), fixed with 4% paraformaldehyde (30 min), washed with PBS (3×), washed with 60% isopropanol, stained with Oil Red (Sigma-Aldrich, UK; 30 min) and finally washed off with 60% isopropanol (1×).

### Chondrogenic differentiation assay

Following trehalose delivery via UMT, MSCs were seeded at a density of 1.6 × 10^7^ cells ml^−1^ in 48-well plates. Micromass cultures were generated by carefully depositing 5 μl of cell solution in the centre of the well with differentiation media (MesenCult™-ACF Chondrogenic Differentiation Kit, StemCell Technologies, Vancouver, BC, Canada), which was replaced every 3 days. After 28 days, the mass formed was washed with PBS (2× ), fixed with 4% paraformaldehyde (30 min), washed with PBS (3×), stained with Safranin-O solution (Sigma-Aldrich, UK; 30 min), washed with PBS (3×), and finally imaged with a contrast-phase microscope. Triplicates were prepared.

### Osteogenic differentiation assay

MSCs were cultured for 2 days, and then UMT-treated for 1 day to allow trehalose internalisation. Subsequently, cells were digested and seeded at 1 × 10^4^ cells ml^−1^ in a 24-well plate until 90% confluency was reached. Osteogenic induction was initiated by replacing the basal media with the osteogenic differentiation media (MesenCult™ Osteogenic Differentiation Kit (Human), StemCell Technologies, Vancouver, BC, Canada). All experiments were carried out in triplicate for different trehalose derivative concentrations alongside negative controls in DMEM only. Osteogenesis was allowed for 14 days with media change every 3 days. Alizarin Red S (ARS; Thermo Scientific, UK) staining was performed to indicate successful osteogenesis of the MSCs by the presence of calcium deposition. Briefly, cells were washed with PBS (3×), fixed with 70% ice-cold ethanol (Sigma-Aldrich, UK) at 4 °C for 1 h followed by ARS staining in the dark for 5 min and 5× washing with PBS (Gibco Life Technologies, Paisley, UK). Images with the stained calcium deposits were taken using a phase-contrast microscope.

### Trehalose internalisation

With the aim of tracking trehalose to validate internalisation into the cell, we labelled trehalose with rhodamine B (ThermoFisher GmbH, Germany). Then, MSCs were treated with rhodamine-labelled trehalose via UMT as previously described and seeded on a glass-bottom dish (Ibidi, Germany). Live imaging using a confocal microscope fitted with an environmental chamber fitted with temperature and CO_2_ controls at 60× was conducted.

### Cell imaging

Cells were imaged using the following range of microscopes, as detailed in their respective methodology section: fluorescence (TiE; Nikon, Japan); confocal (Leica DM500; Leica Microsystems GmbH, Germany) coupled with a CCD camera (MicroPublisher 3.3 RTV, QImaging, Canada), and phase-contrast (Eclipse Ti2; Nikon, Japan) microscopes. 3D reconstruction of confocal microscopy images was done using Imaris (Imaris 10.1.1, Oxford Instruments, Belfast, UK).

### Statistical analysis, reproducibility, & software

Data were analysed using one-way analysis of variance (ANOVA) with Bonferroni’s post hoc test, where *p* values < 0.05 were deemed to be statistically significant. Statistical analysis and graphing were done using GraphPad Prism (version 10.1.0, Dotmatics, Boston, MA, USA). Data are presented as mean values with standard deviations. Relative cell viability refers to the total live cell number of each sample against the average cell number of the control at the specified time point. Illustrations were created using BioRender.com.

### Reporting summary

Further information on research design is available in the Nature Portfolio Reporting Summary linked to this article.

## Results

### Validation: trehalose delivered via UMT does not harm cells

**Fig. 3:**
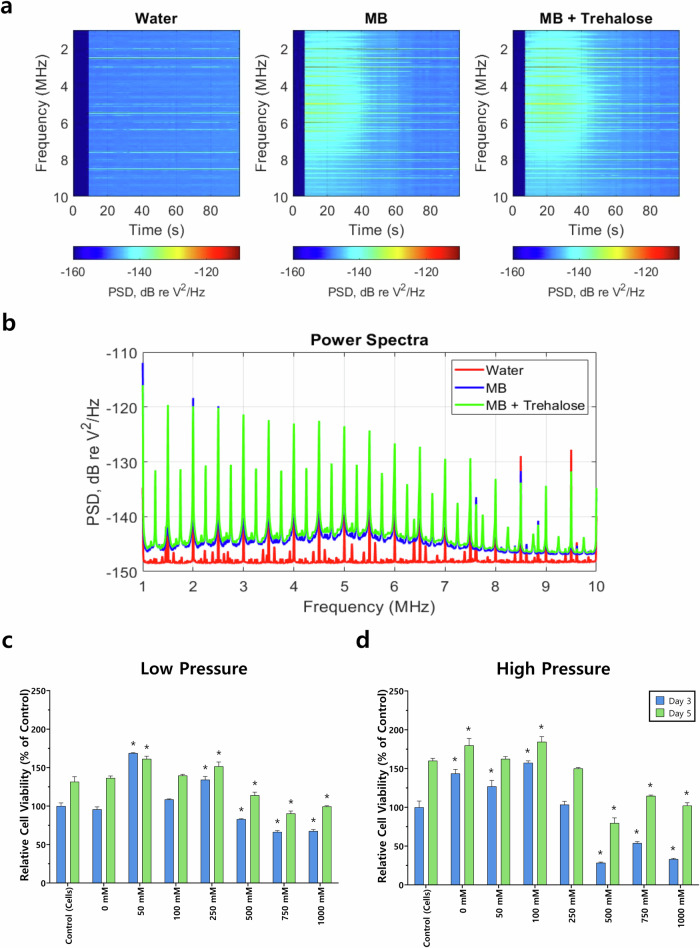
Cavitation signal analysis and fold increase in cell number at 3- and 5-days post-treatment. **a** Spectral density as a function of time for Eppendorf tubes filled with three different media: water (left), SonoVue microbubbles (MB; centre), and microbubbles + trehalose (right). The FUS source is turned on at ~*t* = 7 s. **b** Power spectral densities for the three spectrograms above, averaged over the exposure time. Cells were treated with a range of trehalose concentrations (0–1000 mM) and subjected to either **c** low (0.25 MPa) or **d** high (1.6 MPa) pressure for 5 min. Data are presented as the relative cell viability against the control on day 3. A control of cells not exposed to ultrasonication nor treated with trehalose was also used. Statistically significant differences (*) in comparison with the control are shown (two-way ANOVA with Bonferroni’s post hoc test, *p* < 0.05). Error bars indicate standard deviation (*n* = 3).

**Fig. 4:**
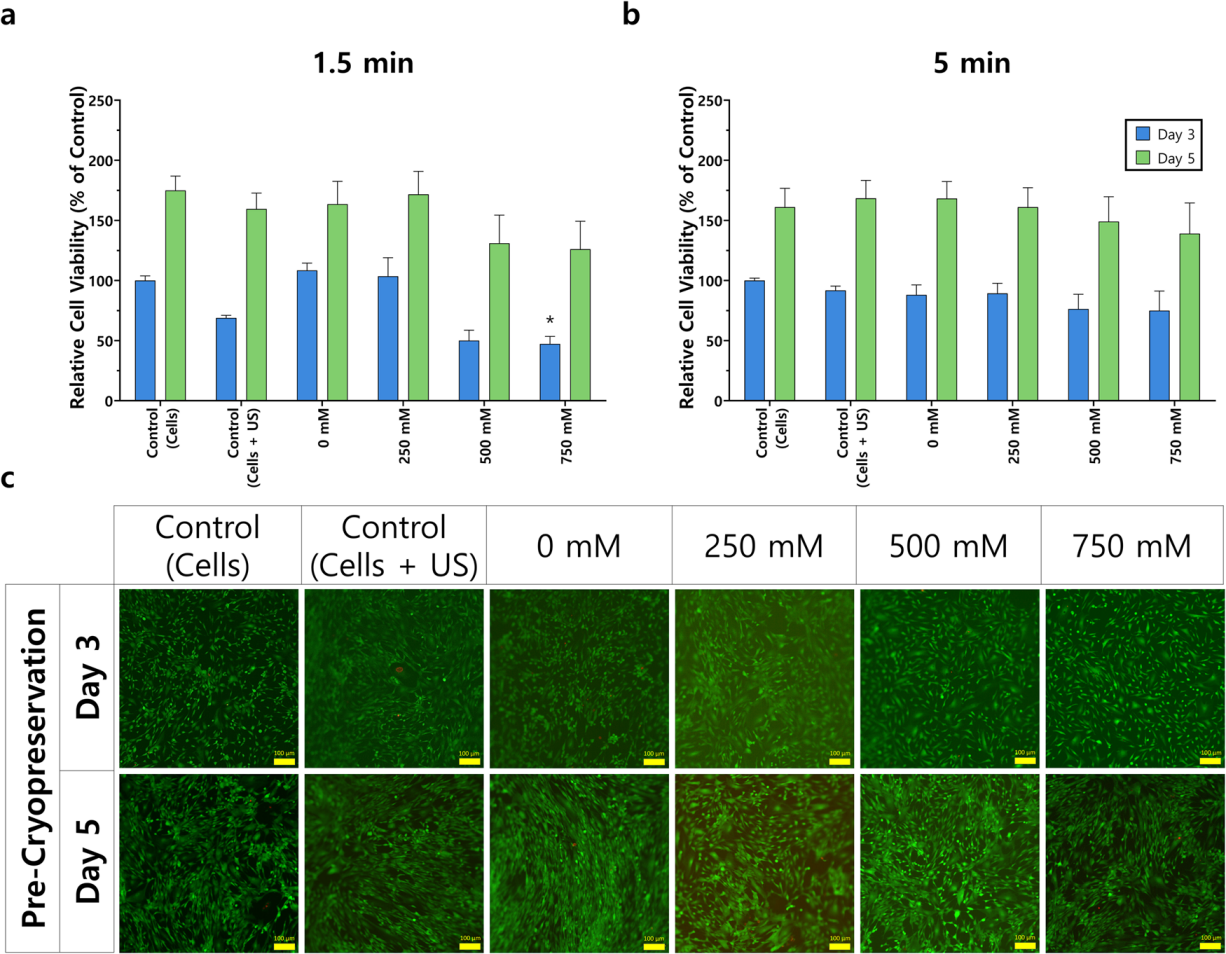
Trehalose-treated MSCs at 3- and 5-days post-treatment and before cryopreservation. Cells were treated with a range of trehalose concentrations (0–750 mM) and subjected to ultrasonication for either **a** 1.5 or **b** 5.0 min at 0.25 MPa. Data are presented as the relative cell viability against the control (cells) on day 3. **c** MSCs were also imaged at these two timepoints and stained with a live/dead assay to depict live (green) and dead (red) cells. Here, cells treated with 5 min UMT are shown. Control (cells): cells not treated with trehalose and not exposed to ultrasonication, and control (cells + US): cells exposed to ultrasonication but not treated with trehalose. Scale bar: 100 μm. Statistically significant differences (*) in comparison with the control (cells) are shown (two-way ANOVA with Bonferroni’s post hoc test, *p* < 0.05). Error bars indicate standard deviation (three independent runs; total *n* = 11).

With the aim of identifying the most suitable parameters for trehalose delivery, we compared a range of trehalose concentrations (0–1000 mM) at low (0.25 MPa) and high (1.6 MPa) US pressure (Fig. 3) as well as exposure times of 1.5 and 5.0 min (Fig. 4). MSCs were treated by UMT and evaluated 3- and 5-days post-treatment, in anticipation of further experimental work involving trehalose treatment followed by cryopreservation.

The assessment of cell viability at days 3 and 5 post-treatment reveals that trehalose concentrations up to 750 mM generally exhibit equivalent values to the control, with concentrations up to 250 mM even more promising. This finding suggests that this concentration range represents the most promising therapeutic window for MSCs.

Moreover, UMT over a range of parameters does not substantially harm cells, as exhibited by the cell viability values obtained when delivering trehalose via UMT to MSCs, which are similar to those of the control. Furthermore, this finding is corroborated by data reported in the literature^30^. In the case of the additional ultrasonication parameters explored, the lower pressure (0.25 MPa) and longer exposure (5 min) yield higher cell viability than their counterparts when comparing the same trehalose concentration. For instance, the average cell viability at day 3 following 500 Mm trehalose concentration delivered via UMT 82.9% (low pressure) vs. 28.5% (high pressure) and 27.1% (1.5 min) vs. 81.7% (5 min) (Figs. 3 and 4). The results suggest that pressures higher than 1.6 MPa would hinder cell viability.

However, the variations of exposure time and pressure explored here do not substantially interfere with the cell viability of MSCs at day 5 post-exposure. The passive cavitation data under these conditions were dominated by harmonic and ultra-harmonic (odd half-integer multiples of the drive frequency) signatures (Fig. 3a, b), suggesting moderate bubble activity that has previously been associated with effective intracellular delivery.

Figure 3a, b shows examples of cavitation signal analysis for Eppendorf tubes containing water (left), SonoVue microbubles (centre), and microbubbles + trehalose (right). In these images, the ultrasound is turned on at ~*t* = 7 s. The signal spectra show a pronounced enhancement of all spectral components when microbubbles are present. Both microbubble samples have elevated ultra-harmonics and broadband noise, both of which are nonlinear emissions associated with microbubble collapse. After ~1.5 min, much of this enhancement has subsided, as expected from gradual microbubble destruction during US exposure. Trehalose had no meaningful effect on the observed cavitation activity.

The comparison between ultrasonication pressures suggests that low pressure is associated with higher cell viability figures than high pressure, which is particularly noticeable when treating cells with higher trehalose concentrations. Furthermore, the data also reveal that the 1000 mM trehalose concentration does not perform as well as its counterparts, particularly when exposed to high pressure. Bearing this in mind, this concentration was discarded from further research.

Upon investigating different exposure times to ultrasonication alongside varying trehalose concentrations, we found that ultrasonication exposure times also play a significant role in cell viability. For instance, UMT at 0.25 MPa for 1.5 min (500 mM, 49.94%; 750 mM, 47.16%) instead of 5 min (500 mM, 76.28%; 750 mM, 74.96%) led to greater variability of viability at day 3, although this distinction was minimal at day 5. A supplement on cavitation as a function of pressure (0.25 and 1.6 MPa) is available in Fig. S3.

The 5-min UMT leads to similar cell viability in comparison with the control (cells), which corroborates that trehalose and ultrasonication treatment, both together (0–750 mM trehalose) and independently (control: cells + US), do not significantly hinder cell viability. This is further validated by microscopy images of stained live and dead cells (Fig. 4) and is an essential finding in the development pipeline of US-delivered trehalose as a CPA, before evaluating post-cryopreservation performance.

### Trehalose delivered via UMT acts as a successful CPA in 4+ weeks cryopreservation

**Fig. 5:**
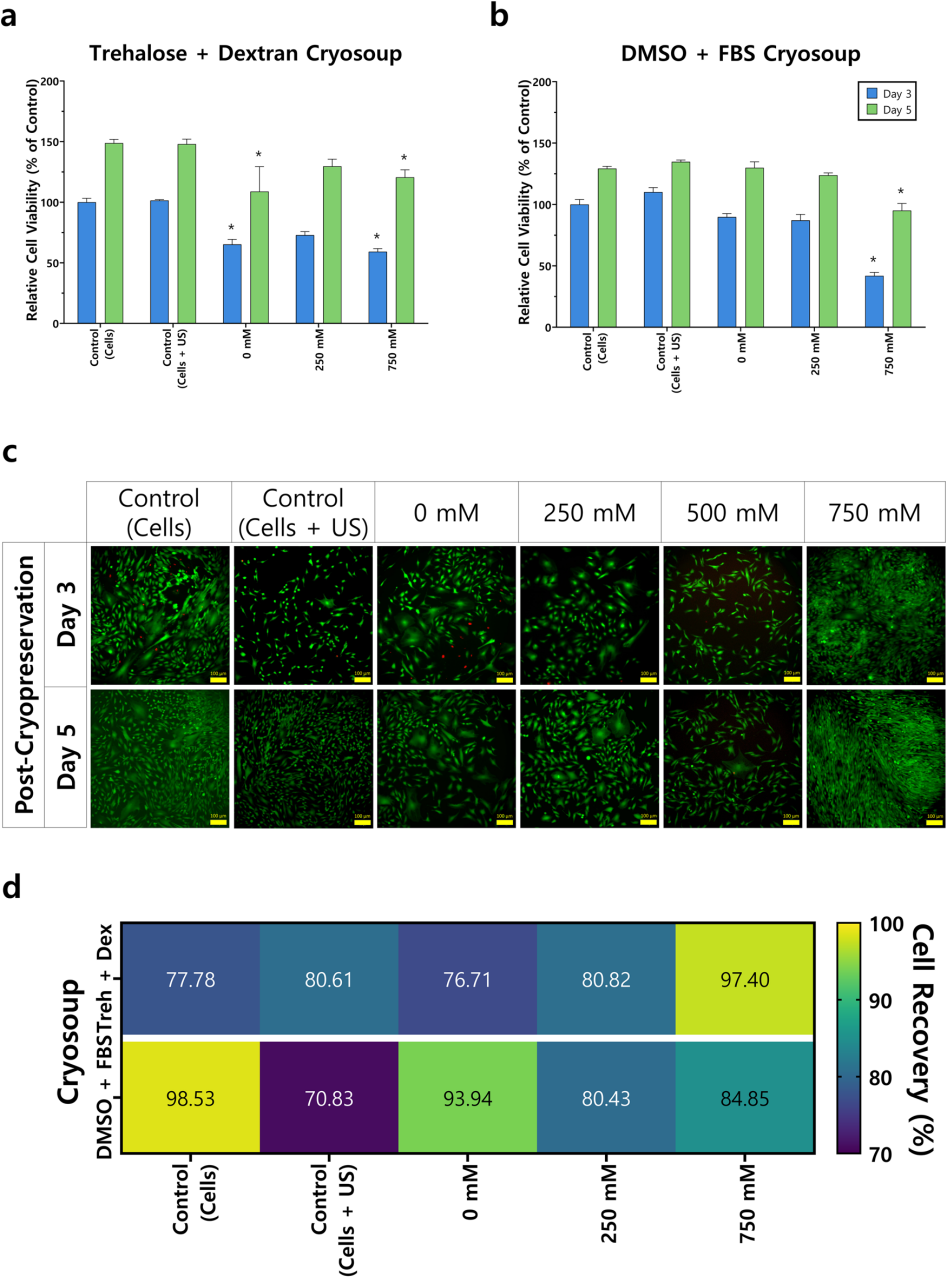
MSCs were treated with US + microbubbles (UMT) (0.25 MPa, 5.0 min) and cryopreserved for at least 4 weeks in liquid nitrogen tanks before seeding. At 3- and 5-days post-seeding, (**a**, **b**) cell viability was assessed (data are presented as the relative cell viability against the control (cells) on day 3) and **c** MSCs were imaged after staining with a live/dead kit. MSCs were cryopreserved using either **a**, **c** 5% trehalose–2.5% dextran or **b** 10% DMSO–90% FBS cryosoups after UMT. **c** Microscopy images depicting live cells (green) and dead cells (red) shown were taken after cryopreservation. Here shown, cells cryopreserved in trehalose–dextran cryosoup. Control (cells): cells not treated with trehalose and not exposed to ultrasonication, and control (cells + US): cells exposed to ultrasonication but not treated with trehalose. Scale bar: 100 μm. Statistically significant differences (*) in comparison with the control (cells) are shown (two-way ANOVA with Bonferroni’s post hoc test, *p* < 0.05). Error bars indicate standard deviation (*n* = 3). **d** Cell recovery calculated as a percentage of cells post- vs. pre-cryopreservation, by cryosoup and trehalose concentration delivered via UMT.

Following the initial assessments of US settings and trehalose concentrations, MSCs were treated by UMT and cryopreserved using two different CPAs (trehalose + dextran vs. DMSO + FBS) for at least 4 weeks in liquid nitrogen tanks, after which cell viability at 3- and 5-days post-seeding was assessed (Fig. 5a, b). It was of interest to compare the use of a trehalose + dextran cryosoup against the conventionally used DMSO + FBS formulation given that most cell therapy applications currently utilise DMSO-based CPAs despite the issues associated with DMSO toxicity^1,5^.

To further validate cell viability and gain insights on cell morphology, MSCs were treated by UMT at a range of concentrations. Cells were assessed both before and after cryopreservation. Using a live/dead assay, cells were stained (live cells: green; dead cells: red) and imaged at 3- and 5-day post-seeding (Fig. 5c). We found that the images obtained support the conclusions drawn from the cell viability quantification addressed before. Also, the morphology of MSCs displayed across the different concentrations of trehalose exhibits the expected spindle-like shape, similar to the control (cells).

When looking at the pre-cryopreservation samples (Fig. 5c), we observe that both the morphology and cell density across cells treated with trehalose at different concentrations and the two controls are similar. This suggests that neither the treatment with trehalose nor the ultrasonication process has a significant negative effect on cell morphology or viability, as exhibited by the similar ratios of live to dead cells depicted in the images.

In the case of the post-cryopreservation samples, we observe that on day 3, there seems to be a lower cell density in contrast with the pre-cryopreservation images. However, we see a notable increase in cell density at day 5 across samples. It is also interesting that in both the controls and the 0 mM trehalose concentration, there is a slightly higher percentage of dead cells. Cell recovery (Fig. 5d) provides additional insights into UMT for cryopreservation. For instance, higher concentrations of trehalose, particularly 750 mM, significantly increase the cell recovery rate in contrast with the 0 mM concentration.

### Trehalose delivered via UMT maintains cell multipotency after cryopreservation

**Fig. 6:**
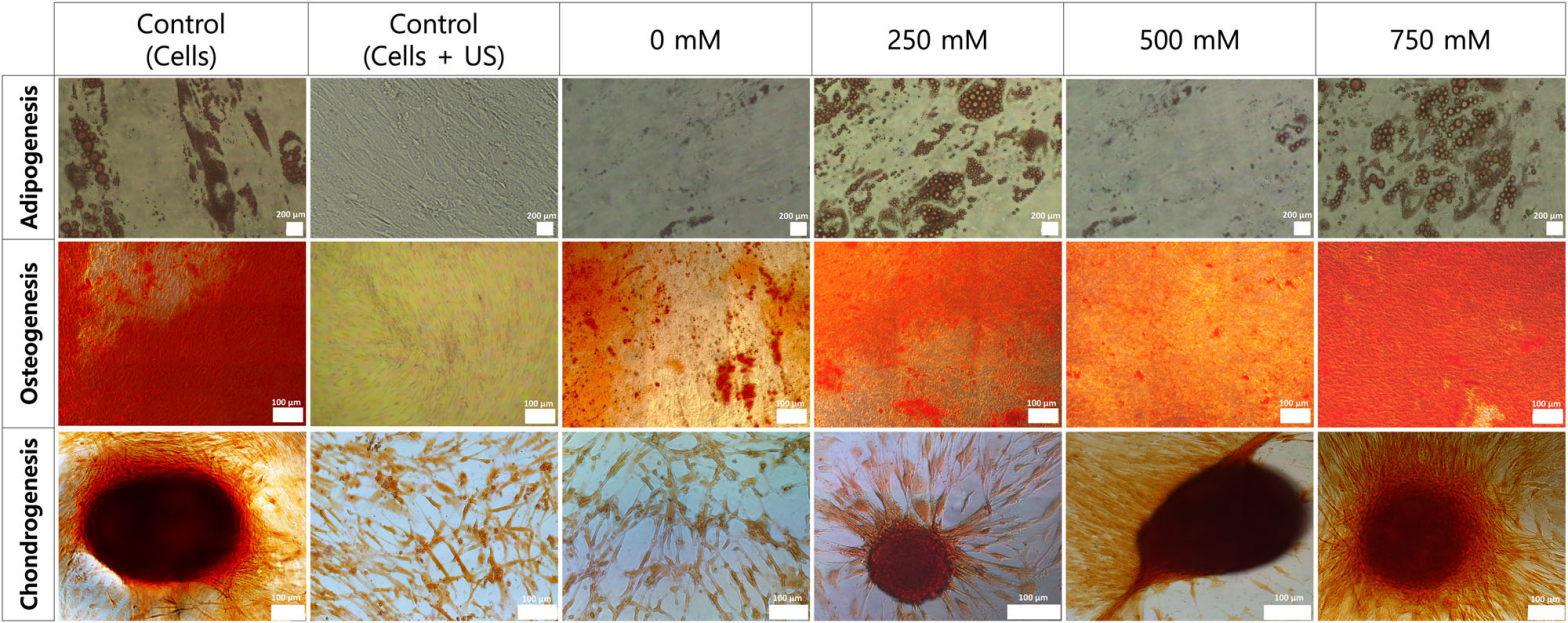
Cell pluripotency post-cryopreservation depicting sample images of cells treated with a range of trehalose concentrations (0–750 mM) delivered via UMT alongside controls. A control of MSCs not exposed to ultrasonication nor treated with trehalose (Control (cells)) and a control of cells only exposed to ultrasonication (0.25 MPa, 5.0 min; control (cells + US)) but not treated with trehalose were used. Controls were cryopreserved in 10% DSMO + 90% FBS to show the difference between DMSO and trehalose as CPA. During the differentiation assay, positive control cells are treated with the differentiation medium while negative controls are given normal medium: DMEM + 10% FBS. Scale bars: 200 μm (adipogenesis), 100 μm (osteogenesis), or 100 μm (chondrogenesis).

Three cell differentiation assays—adipogenesis, osteogenesis, and chondrogenesis—were conducted to validate that pluripotency is maintained after cryopreservation when cells are treated with UMT (Fig. 6). MSCs treated with 250–750 mM of trehalose delivered via UMT show similar differentiation across all three assays (adipogenesis, osteogenesis, and chondrogenesis) compared to the positive control.

For instance, the adipogenesis assay (Fig. 6, top row) helps identify oil droplets inside the cell to validate cell differentiation into adipocytes. These Oil Red-stained droplets are visible in the control (cells) and the UMT-treated MSCs, but not in the control (cells + US) or, to a lesser extent, the ones treated with 0 mM. Interestingly, the 250 and 750 mM trehalose concentrations show a higher concentration of oil droplets than the ones treated with 500 mM.

Calcium deposition stained with Alizarin Red validates osteogenesis cell differentiation into osteocytes. Here (Fig. 6, middle row), we observe this differentiation in all samples except for the control (cells + US) and to a lesser extent in the one treated with 0 mM.

Whereas in chondrogenesis differentiation, the deposition of glycosaminoglycans stained with Safranin-O enables the easy identification of chondrocytes and micropellets (Fig. 6, bottom row), which can be seen in both the control (cells) and the MSCs treated with 250–750 mM of trehalose via UMT. In the chondrogenesis assay, a micromass forms when specific growth factors, essential for inducing cell differentiation into chondrocytes, are present. The negative controls, unlike the positive controls, did not receive any differentiation media and were treated with a normal medium (DMEM + 10% FBS). As expected, no micromass formation occurred in the negative controls. Conversely, the 0 mM group, along with the 250, 500, and 750 mM groups, received a chondrogenic differentiation medium. On the other hand, the control (cells + US) and the 0 mM concentration do not display chondrocyte differentiation. Cells in the 0 mM group could not differentiate into chondrocytes due to hindered pluripotency during the cryopreservation step, as expected when using trehalose to protect cells from physical and mechanical stresses during cryopreservation and then recovery.

The results obtained following trehalose delivered via UMT followed by cryopreservation for at least 4 weeks suggest that utilising trehalose as a CPA and delivering it via ultrasonication successfully preserves cell pluripotency. In contrast, the control that was only exposed to ultrasonication but was not treated with trehalose (negative control) as well as the sample treated with 0 mM of trehalose show differences in cell differentiation, which are particularly noticeable in the adipogenesis and the chondrogenesis assays. Based on the differentiation assays, the most promising trehalose concentrations for UMT treatment are 250 and 750 mM.

### Trehalose internalisation

**Fig. 7:**
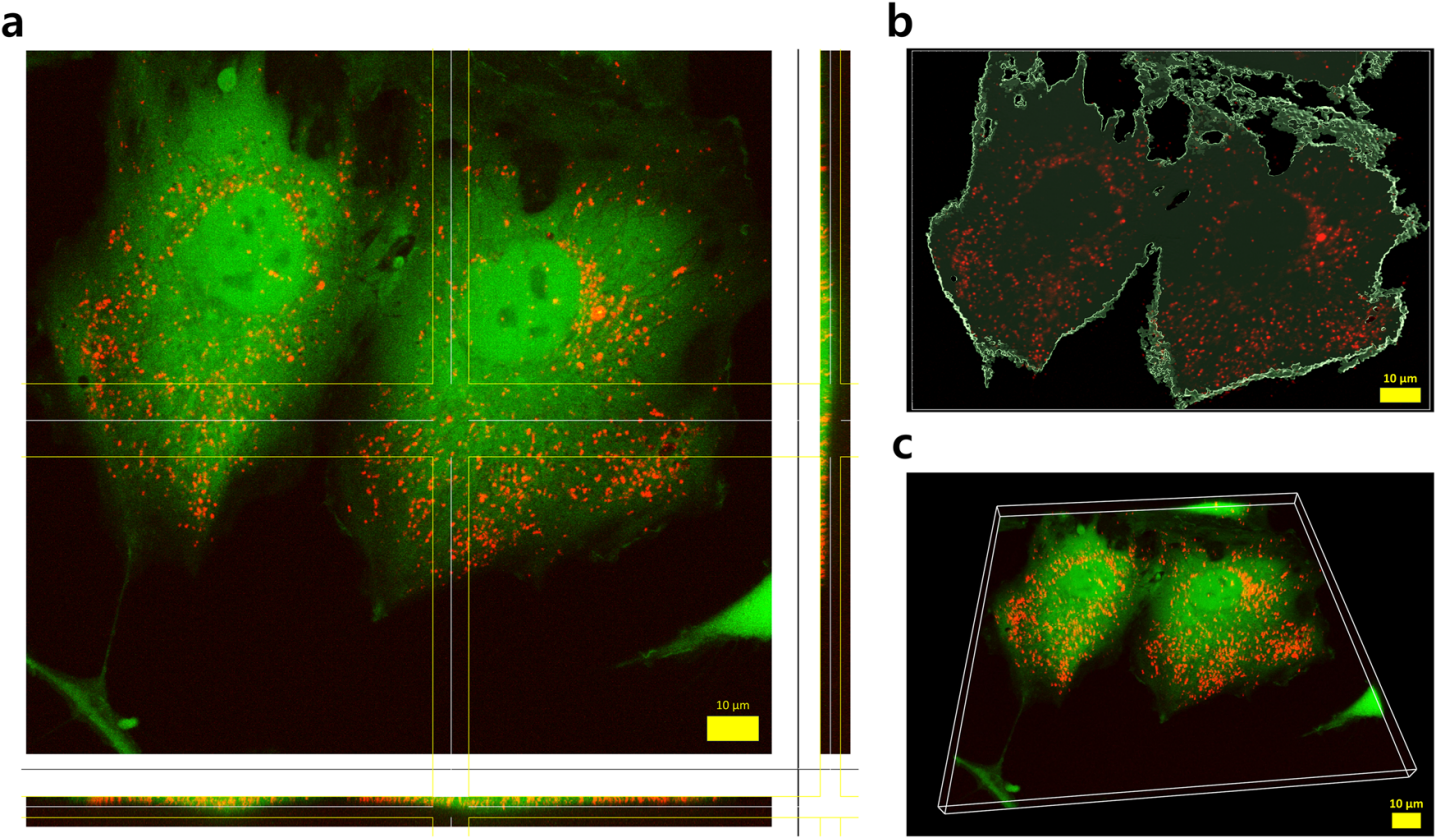
Trehalose internalisation into MSCs achieved via UMT, depicting rhodamine-labelled trehalose (Rhod-Treh; red) and live cells (green). **a** Co-localisation of Rhod-Treh and the cell body. **b** Rhod-Treh mapped within the cell borders. **c** 3D reconstruction from z-stack microscopy images. Images were captured as a z-stack using both green and red channels at a magnification of 60×. Scale bars: 10 μm.

To validate the internalisation of trehalose rather than its presence around the cell, trehalose was fluorescently labelled with rhodamine and delivered via UMT before imaging (Fig. 7 and Fig. S1). The confocal images corroborate the presence of trehalose inside of the cell, reinforcing the hypothesis of the efficiency of UMT as a CPA in providing cryoprotection from inside the cell, potentially contributing to enabling pluripotency function maintenance during cryopreservation.

## Discussion

Trehalose was delivered intracellularly to MSCs using the UMT method, which appeared to overcome conventional barriers to transport across the cell membrane, and yielded viability and differentiation results similar to the controls, all without the toxicity associated with DMSO^1,3,5,7^. As addressed in the results, opting for a low pressure rather than high in the US setup is associated with higher cell viability across the range of trehalose concentrations delivered via UMT; similarly, 5 min of US exposure yields higher cell viability values than 1.5 min, especially when evaluated at 3 days after treatment.

In terms of the ultrasound treatment, the low pressure (0.25 MPa) employed allows for compact and commercially available instrumentation. Exposures were performed with samples in common laboratory vessels, meaning that the proposed method has potential to fit in with a variety of common laboratory workflows. Further acceleration of the treatment process through exposure parameter optimisation and multi-sample parallelisation are subjects of ongoing research.

An unexpected finding was that higher viability and lower variability across trehalose concentrations were associated with a longer US exposure time that well-exceeded the interval where cavitation emissions could be observed. It may be that even after the bubble activity has dissipated, having US-mediated vibration promotes membrane pore closure or otherwise attenuates a lytic process that follows sonoporation. This issue is a matter of ongoing study.

In this work, cells are cryopreserved for at least 4 weeks in liquid nitrogen. Here, the adipogenesis, osteogenesis, and chondrogenesis differentiation assays revealed that MSCs treated with trehalose delivered via UMT maintain cell pluripotency, as shown in Fig. 6. In contrast, the control (cells + US) and the 0 mM concentration do not display the expected cell differentiation.

Various permeating and non-permeating CPAs have been described in previous literature, emphasising the challenge of internalising trehalose. Consequently, trehalose is often encapsulated into different drug-delivery systems^31^ or used in conjunction often used in conjunction with DMSO, despite additional safety concerns for the therapeutic applications^32^. Furthermore, Lomba et al. emphasise the urgency of developing safer alternatives to conventional cryopreservation methods^33^. The importance of secure, reliable, and efficient cryopreservation techniques is of high interest for ensuring safety in therapeutic applications and advancing regenerative medicine. Some studies report storage at −80 °C for just 1 week, which is insufficient to establish the long-term safety of certain CPAs. Also, the differentiation capability of the cryopreserved cells in Buchanan et al.^34^ is evaluated based on the phenotypic analysis of surface markers CD34 (differentiation), CD71 (proliferation), and CD38 (activation) whereas in the work presented here, we validate cell differentiation post-cryopreservation through osteogenesis, chondrogenesis and adipogenesis. Buchanan et al. evaluate cell morphology and cell phenotype post-cryopreservation in DMSO-containing cryosoups. In this research, we opt for a strategy of delivering trehalose both on an intra- and extra-cellular level without the addition of DMSO at any stage. This is a noteworthy advantage, particularly in the context of therapeutic applications for MSCs.

In addition to cell viability and differentiation assessments both pre- and post-cryopreservation, it was essential to validate trehalose internalisation into the cell rather than simply its attachment to the cell membrane. By fluorescently labelling trehalose with rhodamine, it was possible to corroborate the presence of trehalose inside the cell. Given that the presence of a CPA is required on both sides of the cell membrane^3^ to achieve successful cryopreservation, here, we ensure that trehalose was internalised via UMT and also supplied surrounding the cell in the form of a trehalose + dextran cryosoup.

The majority of the studies are focused on delivering trehalose primarily to red blood cells or human platelets, using electroporation^35^, microinjection^36^, and osmotic and thermal shock^11,37^. Unfortunately, most of the current studies involve the use of DMSO during cryopreservation process, a practice that should be avoided in clinical settings. Buchanan et al. developed a genetically engineered poration method, using a self-assembling porating molecule, referred to as H5, which creates 2 nm transmembrane pores that allows the passage of molecules up to 1000 Da. The pore opening can be reversed by the addition of Zn^2+^, which requires chelation agents to remove these ions^11^. While this reversible electroporation proved high cell viability post-treatment, the cells showed normal proliferation rates. The complexity of this method, with its extensive steps prior to application, could impede its therapeutic use.

Further to the cryopreservation efforts addressed in this work, UMT was also utilised to preserve cells during lyophilisation (Fig. S2). Our data show that delivering trehalose via UMT (250, 500, and 750 mM) enables the preservation of the cell membrane post-lyophilisation and without any cell debris. One of the strengths of the current UMT methodology is that it allows for mammalian cells to be successfully cryopreserved and lyophilised. In prior research, erythrocyte preservation is achieved using a microfluidic device, ultrasound, and microbubbles^9^. However, it is important to bear in mind that there exist key differences between erythrocytes and other mammalian cells; for instance, its lack of nucleus and organelles such as mitochondria, which makes membrane permeabilisation easier^11^.

Our results further confirm that trehalose delivered via ultrasonication acts as an effective CPA during both freezing and drying stages of lyophilisation. However, there are some limitations associated with this work. For instance, only MSCs are utilised to validate the feasibility and evaluate the performance of trehalose delivered via UMT as a CPA. While using additional cell lines would be valuable to further optimise the concentrations of trehalose required for UMT treatment, MSCs allow additional validation in terms of preservation of cell function (i.e., cell differentiation).

As for the US setup, additional research on microbubbles type, quantification of ultrasound energy during exposure, and optimisation of other US parameters could help finetune this protocol, particularly bearing in mind the potential for technology transfer to other settings. These microbubbles alternatives would, however, need to be clinically approved for diagnostic purposes to ensure safety for further cell therapy applications, just as the one employed in this research.

We are aware of one prior study using US without microbubbles^23^, where platelets were exposed for 30–60 min. Cavitation was hypothesised as a mechanism, but the protocol did not include any means to observe this effect. If cavitation was the mechanism, then the US exposure would need to be sufficient in amplitude and time scale to first create microbubbles from dissolved gas in the media before stimulating poration in the nearby cells. An advantage to using microbubbles, as shown here and many other studies for delivery into a variety of cell types, is that if the bubbles are already present, the required US amplitude and time scale to achieve the desired bioeffects are reduced substantially.

Also, the sample size could be increased to facilitate the identification of the optimal trehalose concentration rather than a range of concentrations. On a related note, the containers used during the UMT treatment employed here were Eppendorf (2 ml) and cryovials (2 ml). In order to scale up UMT, it would be interesting to assess the impact of opting for containers with a larger volume capacity.

Here we deliver trehalose via ultrasonication to increase the ability of trehalose to internalise into the cell and thus providing protection during freezing and/or drying steps. We investigate the post-cryopreservation and post-lyophilisation outcome impact of the modified trehalose on mammalian cells. We compare as well the cryoprotective effects of the CPAs after cryopreservation and lyophilisation in three model cell lines. Importantly, we demonstrate that counting cells immediately post-thaw can lead to overestimation of cell numbers and the benefit of the CPA. Furthermore, we passage cells to check their pluripotency and their ability to proliferate.

## Supplementary information

Supplementary Information

Reporting Summary

## Data Availability

All data associated with this study are presented in this manuscript. Data are publicly available at https://github.com/carla-fuenteslopez/TrehaloseUS.
