# Supplementary material for: Mesenchymal stem cell cryopreservation with cavitation-mediated trehalose treatment
Source: Commun Eng. 2024 Sep 9;3:129. doi: 10.1038/s44172-024-00265-6 (PMC11385975; doi:10.1038/s44172-024-00265-6)
Supplement: Supplementary file 1 — Supplementary Information [file 44172_2024_265_MOESM1_ESM.pdf]

## Additional information

**Table S1.** **Composition of controls and experimental trehalose concentrations to be delivered by US + microbubbles (UMT).**

| Variation | Formulation | | Treatment | |
|---|---|---|---|---|
| | DMEM | FBS | Trehalose | Ultrasonication |
| Control (Cells) | ✓ | ✓ | × | × |
| Control (Cells + US) | ✓ | ✓ | × | ✓ |
| 0 mM | ✓ | × | × | ✓ |
| 50-1000 mM | ✓ | × | ✓ | ✓ |

**Table S2.** **Composition of cryosoups used for suspending cells during cryopreservation. Both cryosoups used DMEM with either Trehalose-Dextran or DMSO-FBS.**

| Cryosoup | DMSO | FBS | Trehalose in DMEM | Dextran in DMEM |
|---|---|---|---|---|
| Trehalose-Dextran Cryosoup | - | - | 5% | 2.5% |
| DMSO-FBS Cryosoup | 10% | 90% | - | - |

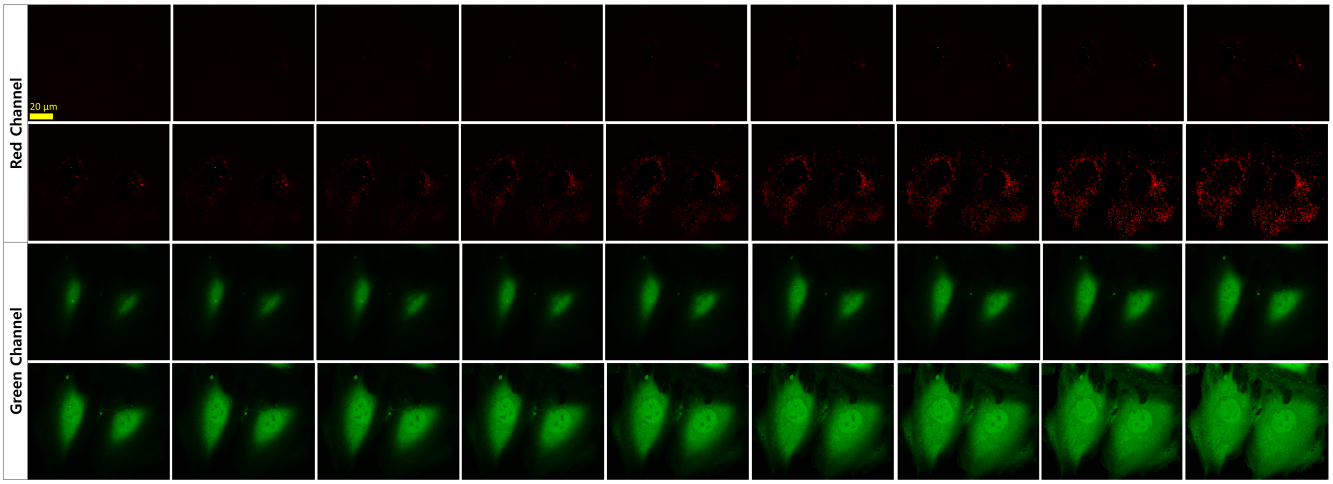


**Figure S1. Optical sectioning of two mesenchymal stem cells (MSCs).** The different images, corresponding to different focal planes, allow one to follow the 3D distribution of the Rhod-Treh (Red Channel) in the cell body of the pigments in the cell body (Green Channel). Scale bar: 20 $\mu$m.

## The Potential of Trehalose as a CPA for Lyophilisation

MSCs treated with trehalose *via* UMT were suspended in 1 ml of trehalose-dextran solution (5% trehalose + 2.5% dextran in DMEM) and deposited in sterilised Teclen® lyophilisation cups (Teclen GmbH, Germany). Samples were then lyophilised (Table S3) with a (Telstar Lyobeta, Spain) before seeding cells for imaging using a fluorescent microscope. Triplicate samples of each experimental (trehalose delivered *via* UMT at a concentration of 250-750 mM) or control (cells not treated with trehalose nor exposed to US) variation were prepared.

Beyond cryopreservation, it was valuable to explore the use of UMT for lyophilisation. To that end, trehalose was delivered UMT at a range of concentrations (250-750 mM) before subjecting the samples to lyophilisation (Table S3). The microscopy images depicting live and dead cells, as well as nuclei (Figure S2), showcase the effect of utilising trehalose as a CPA during lyophilisation. The trehalose concentrations assessed enable cell preservation, as shown by the live cells and nuclei stained in green and blue, respectively, and by the minimal number of dead cells (in red).

**Table S3. Lyophilisation parameters.**

| Lyophilisation Steps | Parameters | | |
|---|---|---|---|
| | Temperature (°C) | Vacuum (mbar) | Time (hours) |
| Freezing | -40 | | 2 |
| Chamber vacuum | | 0.6 | |
| Primary drying | -40 | 0.6 | 20 |
| Primary drying | -20 | 0.6 | 5 |
| Secondary drying | 0 | | 3 |
| Secondary drying | 4 | | 1 |
| Secondary drying | 22 | | 3 |
| End of cycle | | | |

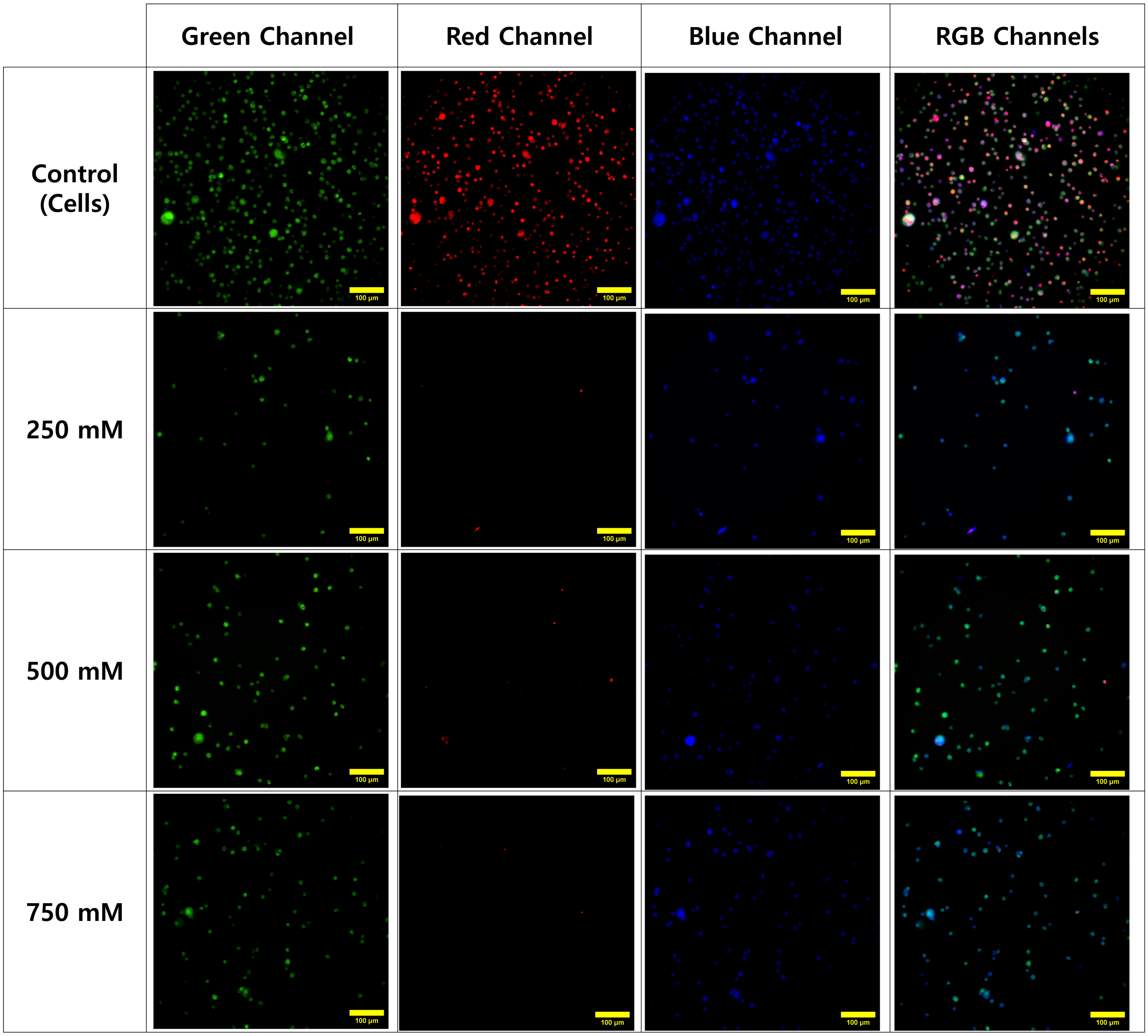


**Figure S2.** **Post-lyophilisation images of MSCs showing an intact cell membrane and nucleus.** Three channels were used to image live (green) and dead cells (red), as well as the nuclei (blue). The channels were then overlapped to create an image depicting all three channels, which prove that delivering trehalose UMT (250, 500 and 750 mM) enables the preservation of the cell membrane post-lyophilisation and without any cell debris. A control (cells) of cells not treated with trehalose delivered UMT was used. Scale bar: 100 $\mu$m.

### Cavitation Data at Two Pressures: 0.25 and 1.6 MPa

Figure S3 shows the power spectral density of cavitation emission signals recorded during five-minute duration exposures with 0.25 (left) and 1.6 (right) MPa peak negative pressures. Both samples contained cells, microbubbles, and trehalose. For the lower pressure, the spectra are primarily tonal (similar to Figure 3a) with emission levels fading into the background noise floor after approximately 90 seconds. By contrast, the 1.6 MPa data set shows its strongest response within the first 10 seconds, with a lower level persisting thereafter. Throughout the exposure, the high-pressure data are characterised by broad, continuous spectrum noise elevations thought to be associated with microbubbles destruction. The availability of bubbles over a longer duration in the high-pressure case is thought to be a consequence of the elevated intensity creating a mixing action in the Eppendorf tube used to contain the sample. Since the tube is larger than the US beam volume, there is a tendency to cause a recirculation flow that introduces a small number of unexposed microbubbles or coalesced gas from previously destroyed microbubbles into the US focal region.

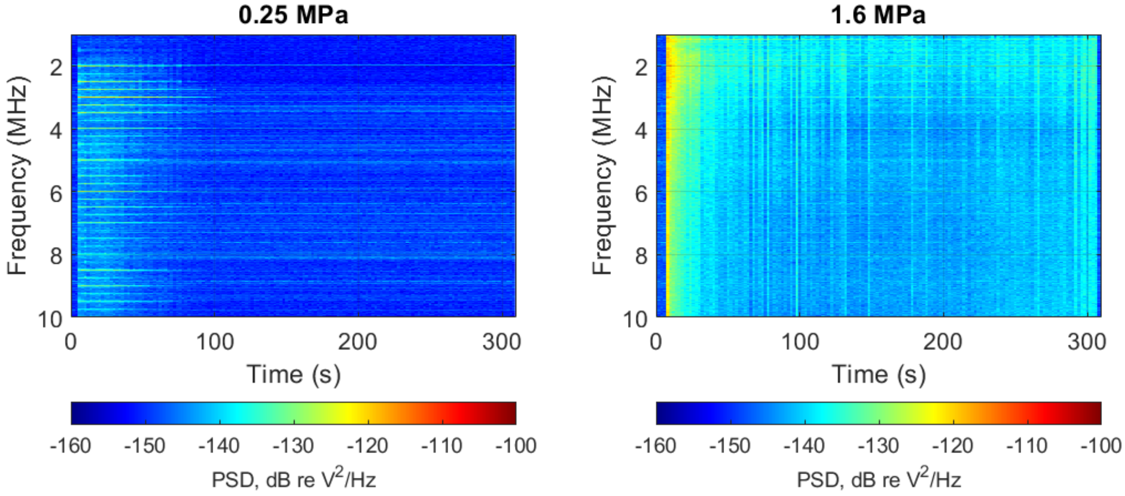


**Figure S3. Cavitation signal power spectral density as a function of time for 0.25 (left) and 1.6 (right) MPa peak negative pressures.** Identical sample compositions of cells, microbubbles, and trehalose were used for both data sets. The US source is turned on at ~t = 5 seconds.
