# Supplementary material for: Mesenchymal stem cell cryopreservation with cavitation-mediated trehalose treatment
Source: Commun Eng. 2024 Sep 9;3:129. doi: 10.1038/s44172-024-00265-6 (PMC11385975; doi:10.1038/s44172-024-00265-6)
Supplement: Supplementary file 2 — Reporting Summary [file 44172_2024_265_MOESM2_ESM.pdf]

nature portfolio

Corresponding author(s): Viorica Patrulea
Hua Ye

Last updated by author(s): Jul 20, 2024

Reporting Summary

Nature Portfolio wishes to improve the reproducibility of the work that we publish. This form provides structure for consistency and transparency in reporting. For further information on Nature Portfolio policies, see our Editorial Policies and the Editorial Policy Checklist.

Statistics

For all statistical analyses, confirm that the following items are present in the figure legend, table legend, main text, or Methods section.

n/a	Confirmed	
☐	☒	The exact sample size (*n*) for each experimental group/condition, given as a discrete number and unit of measurement
☐	☒	A statement on whether measurements were taken from distinct samples or whether the same sample was measured repeatedly
☐	☒	The statistical test(s) used AND whether they are one- or two-sided<br>*Only common tests should be described solely by name; describe more complex techniques in the Methods section.*
☐	☒	A description of all covariates tested
☐	☒	A description of any assumptions or corrections, such as tests of normality and adjustment for multiple comparisons
☐	☒	A full description of the statistical parameters including central tendency (e.g. means) or other basic estimates (e.g. regression coefficient) AND variation (e.g. standard deviation) or associated estimates of uncertainty (e.g. confidence intervals)
☐	☒	For null hypothesis testing, the test statistic (e.g. *F*, *t*, *r*) with confidence intervals, effect sizes, degrees of freedom and *P* value noted<br>*Give P values as exact values whenever suitable.*
☒	☐	For Bayesian analysis, information on the choice of priors and Markov chain Monte Carlo settings
☒	☐	For hierarchical and complex designs, identification of the appropriate level for tests and full reporting of outcomes
☒	☐	Estimates of effect sizes (e.g. Cohen's *d*, Pearson's *r*), indicating how they were calculated

Our web collection on statistics for biologists contains articles on many of the points above.

Software and code

Policy information about availability of computer code

Data collection	Mendeley (online version) and Micrsoft(R) Excel(R) for Microsoft 365 MSO (version 2406 Build 16.0.17726.20078) 64-bit
Data analysis	Statistical analysis were performed in GraphPad (version 10.1.0)

For manuscripts utilizing custom algorithms or software that are central to the research but not yet described in published literature, software must be made available to editors and reviewers. We strongly encourage code deposition in a community repository (e.g. GitHub). See the Nature Portfolio guidelines for submitting code & software for further information.

Data

Policy information about availability of data

All manuscripts must include a data availability statement. This statement should provide the following information, where applicable:

- Accession codes, unique identifiers, or web links for publicly available datasets
- A description of any restrictions on data availability
- For clinical datasets or third party data, please ensure that the statement adheres to our policy

All data associated with this study is presented in this manuscript. Data is publicly available at https://github.com/carlafuenteslopez/TrehaloseUS.
Composition of controls and experimental trehalose concentrations to be delivered by US + microbubbles is supplied in the Supplementary Materials as Table S1; Composition of cryosoups used for suspending cells during cryopreservation can be found in Table S2 and lyophilisation parameters for MSCs are included in Table

S3.
Optical sectioning of two mesenchymal stem cells (MSCs) corresponding to different confocal planes is included in the Supplementary Materials (Figure S1); Post-lyophilisation images of MSCs showing an intact cell membrane and nucleus are in Figure S2, and Power Spectra Density of cavitation emission signals as a function of time for 0.25 and 1.6 MPa peak negative pressures are in Figure S3.

Research involving human participants, their data, or biological material

Policy information about studies with human participants or human data. See also policy information about sex, gender (identity/presentation), and sexual orientation and race, ethnicity and racism.

Reporting on sex and gender	Not applicable
Reporting on race, ethnicity, or other socially relevant groupings	Not applicable
Population characteristics	Not applicable
Recruitment	Not applicable
Ethics oversight	Not applicable

Note that full information on the approval of the study protocol must also be provided in the manuscript.

Field-specific reporting

Please select the one below that is the best fit for your research. If you are not sure, read the appropriate sections before making your selection.

☒ Life sciences ☐ Behavioural & social sciences ☐ Ecological, evolutionary & environmental sciences

For a reference copy of the document with all sections, see nature.com/documents/nr-reporting-summary-flat.pdf

Life sciences study design

All studies must disclose on these points even when the disclosure is negative.

Sample size	Due to the high reproducibility and consistency between cell cultures, sample size of at least n=3 would allow for adequate analysis to reach meaningful conclusions of the data.
Data exclusions	No data were excluded from the analysis
Replication	To ensure robust reproducibility of our data, experiments were run at least in triplicata and different days. All attemps at replication were successful.
Randomization	Randomisation was not applicable for our analysis.
Blinding	Experimental blinding was not possible as the Investigators were aware of the experimental groups. All data collected was quantifiable and blinding would not change any bias in data collected.

Reporting for specific materials, systems and methods

We require information from authors about some types of materials, experimental systems and methods used in many studies. Here, indicate whether each material, system or method listed is relevant to your study. If you are not sure if a list item applies to your research, read the appropriate section before selecting a response.

Materials & experimental systems

n/a	Involved in the study	
☒	☐	Antibodies
☐	☒	Eukaryotic cell lines
☒	☐	Palaeontology and archaeology
☒	☐	Animals and other organisms
☒	☐	Clinical data
☒	☐	Dual use research of concern
☒	☐	Plants

Methods

n/a	Involved in the study	
☒	☐	ChIP-seq
☒	☐	Flow cytometry
☒	☐	MRI-based neuroimaging

Eukaryotic cell lines

Policy information about cell lines and Sex and Gender in Research

Cell line source(s)	Human mesenchymal stem cells (TERT+ mesenchymal cells), immortalised as described by Mihara, et al. (2003), were a gift from the Department of Paediatrics and Adolescent Medicine, LKS Faculty of Medicine, The University of Hong Kong.
Authentication	TERT expression was detected by reverse transcription polymerase chain reaction (RT-PCR) and by immunofluorescence staining with a hTERT-specific rabbit antibody performed by LKS Faculty of Medicine, The University of Hong Kong.
Mycoplasma contamination	All cell lines were tested and confirmed negative for mycoplasma.
Commonly misidentified lines (See ICLAC register)	No commonly misidentified lines were used.

Plants

Seed stocks	No seed stocks were used in this study.
Novel plant genotypes	No novel plants genotypes were used in this study.
Authentication	Not applicable.
